# Direct Oral anticoagulants in pulmonary thromboembolism therapy: the generalization-individualization dilemma and the path to risk stratification—a perspective

**DOI:** 10.3389/fmed.2025.1708409

**Published:** 2025-12-01

**Authors:** Cheng Meng, Hao Wang

**Affiliations:** 1 First Ward of Respiratory and Critical Care Medicine Department, Yan’an People’s Hospital, Yan’an, China; 2 Second Ward of Respiratory and Critical Care Medicine Department, Yan’an People’s Hospital, Yan’an, China

**Keywords:** direct oral anticoagulants, risk stratification, personalized medicine, treatment individualization, anticoagulation therapy, real-world evidence, pulmonary thromboembolism

## Abstract

Direct oral anticoagulants (DOACs) have transformed the management of pulmonary thromboembolism (PTE), supported by robust evidence from phase III clinical trials and subsequent incorporation into international treatment guidelines. However, their fixed-dose convenience and simplified administration have also encouraged a trend toward generalized use in real-world practice, often overlooking essential individual factors such as comorbidities, dynamic physiological parameters, and specific clinical contexts. This perspective synthesizes current evidence and clinical insights from a narrative review of recent literature on the use of DOACs in PTE. It examines the factors promoting their broad application, identifies patient-specific risk profiles, and proposes a framework for dynamic risk stratification to guide personalized treatment. Future efforts should focus on developing and validating integrated stratification systems that incorporate clinical phenotypes, biomarker trends, and patient preferences. Such tools are essential to achieving truly personalized anticoagulation therapy, ultimately maximizing safety and efficacy for each individual with PTE.

## 1 Introduction

### 1.1 Revolutionary simplification in treatment

Direct oral anticoagulants (DOACs), such as rivaroxaban and apixaban, represent a significant advancement in the management of venous thromboembolism (VTE) (1, 2). Compared to traditional vitamin K antagonists (e.g., warfarin), DOACs offer advantages including fixed dosing, no requirement for routine coagulation monitoring, fewer drug interactions, and a rapid onset of action (1, 2). These benefits substantially reduce the complexity and management burden of anticoagulation therapy, improving patient adherence and quality of life (3). Based on consistent and superior results from multiple large Phase III clinical trials, international guidelines now recommend DOACs as first-line treatment for acute pulmonary thromboembolism (PTE) and long-term management (3, 4). Thus, DOACs have become a cornerstone of modern PTE treatment, achieving a “de-complexification” of anticoagulation management (3).

### 1.2 Emergence of a generalization trend

With the widespread adoption of DOACs, a trend toward “generalization” in clinical practice has gradually emerged (5). While their convenience improves treatment accessibility, it has also led some clinicians to adopt a “diagnose-and-prescribe” approach (5). DOACs are increasingly being regarded not merely as an “evidence-based preferred option” but as a “universal solution” applicable to the vast majority of PTE patients (6). Although this trend may align with efficiency demands, it risks overlooking individual patient differences, particularly in those with comorbidities or special clinical contexts (7).

### 1.3 Core paradox and clinical dilemma

A significant contradiction has emerged between the “standardized” treatment model facilitated by DOACs and the considerable “heterogeneity” of PTE patients (8). The generalized application of DOACs may obscure the need for individualized decision-making in patients with complex comorbidities or special clinical contexts (9–12). Uncritical reliance on a “one-size-fits-all” strategy risks of adverse outcomes and clinical oversights, a concern stemming in part from the extrapolation of evidence from idealized trial populations to real-world practice (13).

### 1.4 Study framework and objectives

Within this context, this perspective aims to critically analyze the drivers and potential risks of the generalized use of DOACs in PTE treatment; evaluate the limitations of current risk assessment tools; and propose a theoretical framework and practical pathway for developing a next-generation risk stratification system. By emphasizing the importance of individualized therapy, this perspective argues that the pursuit of treatment convenience should not come at the expense of precision in patient management, thereby promoting safer and more rational use of DOACs in real-world clinical practice.

### 1.5 Methodological approach

This perspective was developed through a narrative review and synthesis of the current literature on DOACs in PE. A comprehensive literature search was conducted utilizing the PubMed and Web of Science databases for publications from 2010 to 2025, employing key search terms such as “direct oral anticoagulants,” “pulmonary embolism,” “risk stratification,” and “real-world evidence.” The analysis prioritized high-impact evidence, including clinical practice guidelines, pivotal randomized controlled trials, large-scale observational studies, and systematic reviews. Identified literature was analyzed thematically to distill prevailing challenges and emergent themes regarding the generalization of DOACs therapy. The ensuing argument is structured in a three-part sequence: a critical examination of the drivers behind the broad application of DOACs; an analysis of the clinical risks arising from insufficient therapy individualization; and the proposal of a conceptual framework for dynamic risk-adapted management.

## 2 Drivers and real-world manifestations of generalized application

### 2.1 Drivers of generalization: evidence, guidelines, and clinical heuristics

The trend toward broadening DOACs use is propelled by several interconnected factors. First, the foundational evidence for DOACs comes from randomized controlled trials (RCTs) involving highly selected populations, which frequently excluded patients with severe renal impairment, active cancer, frailty, or extreme body weight (13–15). This inherent limitation introduces considerable uncertainty when applying RCTs findings to the complex, often “atypical” patients commonly seen in real-world clinical practice (16, 17). Second, although international guidelines strongly recommend DOACs, their detailed recommendations are often simplified into standardized protocols in busy clinical environments. This simplification can dilute the emphasis on individualized patient considerations that are typically underscored in the original guidelines (18, 19). Finally, the fixed dosing and lack of required routine monitoring—key practical advantages of DOACs—can promote cognitive shortcuts. This may encourage a “diagnose-and-prescribe” approach among clinicians. This efficiency-driven practice may reduce the impetus for a thorough, patient-specific risk assessment (13, 20–22). Collectively, the extrapolation of trial evidence, the simplification of guidelines, and the appeal of clinical convenience generate a powerful impetus for broad DOACs use.

### 2.2 Generalized DOACs use in clinical practice: risk management gaps and mitigation needs

In real-world practice, the generalized application of DOACs is evident in their routine use for patients in complex clinical scenarios, often without adequate consideration of subgroup-specific risks or the implementation of dynamic risk assessment strategies (23, 24). Furthermore, dynamic risk assessment—including periodic renal function reevaluation, bleeding event assessment, and treatment duration reconsideration—is often overlooked in patients on long-term anticoagulation (25). These practices highlight critical gaps in current DOACs management that necessitate more structured risk-stratification tools for mitigation.

## 3 Three-dimensional concerns of missing individualization: clinical risks and decision-making blind spots

### 3.1 Therapeutic uncertainties and risk heterogeneity in special populations

The limitations of a generalized DOACs approach are most evident precisely in key patient subgroups who are poorly represented in pivotal RCTs. The management of special populations presents significant challenges in the generalized use of DOACs. Patients with active cancer face concurrently high risks of both thrombotic recurrence and bleeding, often alongside complex medication regimens involving strong inhibitors or inducers of CYP3A4 and P-glycoprotein, which can markedly alter DOACs plasma concentrations and compromise efficacy and safety (26, 27). Therefore, selecting among specific DOACs (e.g., apixaban or rivaroxaban) in this population requires particularly careful consideration (27). Patients at extreme body weights (including both obesity and low body weight) exhibit significant pharmacokinetic variability; standard dosing may lead to under-exposure or accumulation, increasing the risk of recurrent thrombosis or bleeding (28, 29). Elderly, frail, and renal insufficiency patients experience declining physiological reserve, fluctuating creatinine clearance, high fall risk, polypharmacy, and notably elevated bleeding risk (10). Moreover, patients with antiphospholipid syndrome, particularly those who are triple-positive, may face a higher risk of recurrent thrombosis with certain DOACs (30). These scenarios underscore the safety limitations of generalized DOACs application and highlight the necessity for tailored management strategies.

### 3.2 Ambiguity in long-term anticoagulation decisions and limitations of predictive tools

The decision to extend anticoagulation beyond the initial treatment period in patients with PTE is critical yet complex (31). In an ideal setting, only those with high recurrence risk and low bleeding risk should receive indefinite anticoagulation (32). However, accurate tools to identify this subgroup are currently lacking. Existing clinical prediction models, such as VTE-BLEED and HERDOO2, were largely developed in warfarin-treated cohorts, and their validity and calibration in DOACs-treated populations have not been sufficiently established (31, 33). These tools may fail to adequately capture DOACs-specific risk profiles—for example, DOACs’ relatively lower intracranial hemorrhage risk might shift traditional benefit–risk thresholds (34). This evidence gap often forces clinicians to rely on empirical judgment rather than quantitative tools, introducing subjectivity and inconsistency into long-term treatment decisions.

### 3.3 Hidden gaps in follow-up and weakened risk management

The convenience of DOACs largely stems from their fixed dosing without routine coagulation monitoring; however, this advantage has also led to the misconception that “no monitoring” equates to “no management” (35, 36). In real-world practice, this misinterpretation can result in the omission of critical follow-up components. For example, medication adherence—a key factor in anticoagulation failure—is difficult to track objectively (37). Additionally, dynamic changes in renal function (particularly in patients with moderate to severe renal impairment) necessitate at least annual reassessment of creatinine clearance, yet this monitoring is often overlooked in busy outpatient settings (38). Furthermore, patients may newly use over-the-counter medications or be prescribed additional drugs (e.g., antiplatelet agents, NSAIDs, or certain antibiotics) that have potential interactions with DOACs, yet systematic screening mechanisms for such interactions are frequently absent (39, 40). Therefore, without structured follow-up and proactive risk monitoring systems, the current DOACs management paradigm may be insufficient to comprehensively safeguard long-term treatment safety.

## 4 Breaking the deadlock: toward a next-generation comprehensive risk stratification system

**Figure 1:**
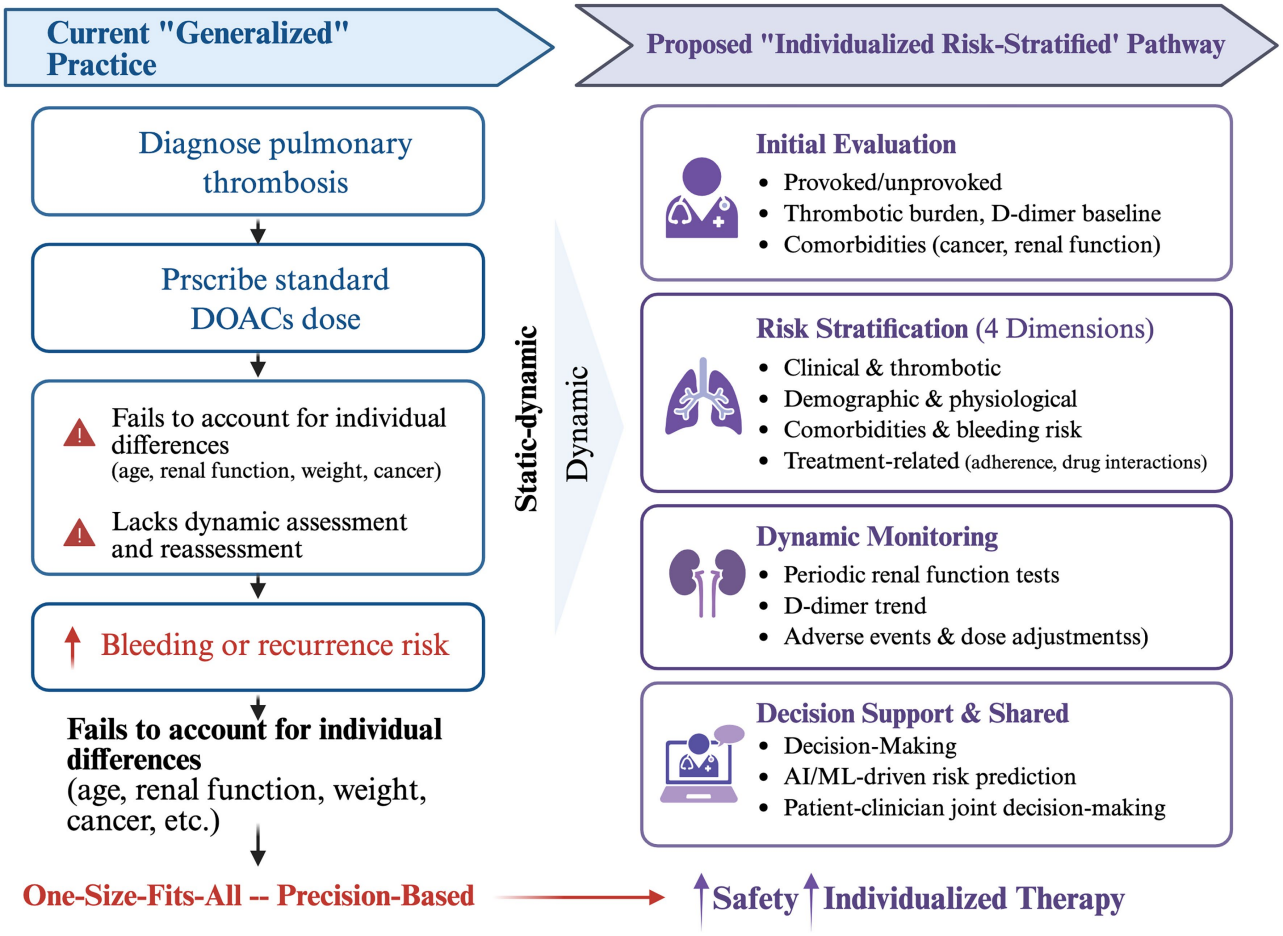
From generalized to individualized pathway in DOACs therapy for pulmonary thrombosis.

The current challenges in DOACs therapy for PTE, along with the proposed pathway toward a more personalized strategy, are summarized in Figure 1. This framework visually articulates the necessary shift from a uniform treatment paradigm to a multidimensional, dynamically adaptive risk stratification model.

### 4.1 Critique and reflection on existing risk stratification models

Currently available risk assessment tools for guiding anticoagulation decisions in PTE demonstrate significant limitations. Most of these models were developed from historical cohorts, particularly during the warfarin era, and their predictive variables are restricted to narrow dimensions—often focusing solely on bleeding or recurrent thrombosis (41). More critically, these tools are essentially static models, classifying patients based on baseline characteristics at a single time point (42). Such an approach fails to capture the dynamic evolution of patients’ clinical status in real-world practice. The reliance on static, one-dimensional modeling substantially undermines their utility in informing individualized treatment strategies (43).

### 4.2 Core principles of a next-generation stratification system

To overcome these limitations, the next-generation stratification framework should adhere to three guiding principles. First, the multi-dimensional principle requires integration of diverse information sources, including features of the index event (e.g., provoking factors, thrombus burden), patient demographics and physiological parameters, dynamic clinical indicators, comorbidities, and psychosocial determinants influencing treatment preferences and adherence (44, 45). Second, the dynamic principle emphasizes the capacity for repeated reassessment, enabling risk reclassification in response to clinical changes such as bleeding events, progressive renal impairment, or remission and progression of malignancy (41, 45). Third, the integrative principle highlights the incorporation of objective biomarkers (e.g., serial D-dimer trends), imaging findings (e.g., residual thrombus evaluation), and patient-centered values and preferences into decision-making, thereby promoting truly shared decision-making (41, 44).

### 4.3 Proposed dimensional framework for risk stratification

Based on these principles, we propose a framework encompassing four core dimensions. Dimension 1: Index event and thrombus biology, including provoked versus unprovoked status, initial thrombus burden, and baseline and serial D-dimer measurements (46, 47). Dimension 2: Demographic and physiological parameters, covering age, body mass index, renal function (with creatinine clearance as a key marker), hepatic function, and other physiological determinants of drug metabolism and clearance (46, 48). Dimension 3: Comorbidities and complications, focusing on active cancer, cardiovascular disease, antiphospholipid syndrome (particularly triple-positive cases), prior bleeding history, and current bleeding risk (46, 47). Dimension 4: Treatment-related factors, including the initial and subsequent choice of anticoagulants, patient adherence, potential drug–drug interactions, and shared expectations between patients and clinicians regarding treatment duration (46, 47).

### 4.4 Technological empowerment and future directions

Artificial intelligence and machine learning (ML) offer a viable pathway for building and operationalizing this complex stratification system (49–51). These technologies enable efficient processing of multi-source, high-dimensional, and unstructured healthcare data, uncovering complex patterns and interactions that are difficult to detect by human analysis (49, 50). Through ML algorithms, dynamic and individualized risk prediction models can be developed to more accurately estimate both recurrence and bleeding risks (49–51). Ultimately, such models may be embedded within clinical decision support systems, delivering real-time, evidence-based risk stratification and treatment recommendations at the point of care (50, 51). This integration will help translate the vision of precision medicine into routine clinical practice.

## 5 Implementation challenges and future research directions

### 5.1 Practical challenges and feasibility concerns in clinical translation

Although the proposed next-generation risk stratification system offers clear theoretical advantages, its clinical translation faces multiple substantive barriers. The first challenge lies in striking a balance between model complexity and clinical usability—highly sophisticated multivariable models may yield superior accuracy but are difficult to apply efficiently in busy outpatient settings (52, 53). Second, the framework requires seamless integration with existing electronic health record systems, which raises issues of data standardization, interoperability, and privacy protection, as well as organizational and administrative hurdles (52, 53). Third, changing physicians’ entrenched diagnostic and therapeutic behaviors is inherently challenging and will require targeted education and training to foster behavioral adaptation (52). Finally, rigorous health economic evaluations are indispensable to determine whether the system is cost-effective while improving clinical outcomes, as this directly influences its potential to secure policy endorsement and financial support (54).

### 5.2 Future research agenda and evidence-generation pathways

To address current evidence gaps and advance the field, future research should prioritize several directions. The foremost priority is to initiate large-scale, prospective real-world studies and registry-based investigations targeting special populations such as patients with extreme body weight, active malignancy, or multiple comorbidities, in order to generate robust data on the safety and effectiveness of DOACs in these groups (21, 28). As a concrete step, we plan to initiate a prospective cohort study to apply our proposed risk-stratification framework. This study will focus on patients with cancer-associated thrombosis and extreme body weight, with the explicit aim of providing real-world validation for the individualized anticoagulation strategy. Second, dedicated efforts are required to develop, validate, and externally calibrate novel risk prediction models tailored for DOACs, ensuring both high discriminatory power and clinical utility (55). In parallel, research should explore the potential role of pharmacokinetic-guided dosing strategies in populations with marked pharmacokinetic variability—for example, individuals with severe obesity or rapidly declining renal function—to provide an evidence base for precision dosing (21, 55).

### 5.3 Implications for guideline development and health policy

This conceptual framework has direct implications for future clinical guidelines and healthcare policy. Guideline committees should consider incorporating multi-dimensional, risk-adapted recommendations, progressively replacing the conventional “one-size-fits-all” approach, thereby formally endorsing precision anticoagulation in authoritative clinical documents (56, 57). At the same time, payers and policymakers should prioritize funding and supporting health technology assessments to comprehensively evaluate the clinical effectiveness, cost-effectiveness, equity considerations, and implementation feasibility of such stratification systems (56–58). These evaluations will provide the critical evidence base required for widespread adoption and eventual inclusion into healthcare reimbursement schemes.

## 6 Summary

### 6.1 DOACs: advances in therapy amid persistent individualized risks

DOACs represent a major advancement in anticoagulant therapy for PTE, markedly improving convenience and safety. However, their widespread adoption carries the risk of overlooking patient heterogeneity, particularly in special populations and due to evidence gaps, potentially increasing bleeding or recurrence risks.

### 6.2 Risk stratification: a necessary path toward precision anticoagulation

The development of a comprehensive and dynamic risk stratification system is essential to address current challenges and to optimize the balance between therapeutic efficacy and safety. Such a system should integrate clinical characteristics, biomarkers, real-time physiological parameters, and patient preferences, thereby enabling treatment decisions that are both precise and individualized.

### 6.3 Multidisciplinary collaboration and future outlook

Achieving this vision requires close collaboration across disciplines, including clinical medicine, pharmacy, informatics, and epidemiology. Through continuous evidence generation, technological integration, and real-world feedback, the application of DOACs in PTE management can be refined to achieve precision anticoagulation, ultimately ensuring that every patient derives safe and effective benefit.

## Data Availability

The original contributions presented in the study are included in the article/supplementary material, further inquiries can be directed to the corresponding author.
